# Medial Open-Wedge High Tibial Osteotomy with Partial Meniscectomy and Without Cyst Excision for Popliteal Cysts: A Case Series

**DOI:** 10.3390/biomedicines13010215

**Published:** 2025-01-16

**Authors:** Kang-Il Kim, Jun-Ho Kim

**Affiliations:** 1 Department of Orthopaedic Surgery, Center for Joint Diseases, Kyung Hee University Hospital at Gangdong, Seoul 05278, Republic of Korea; 2 Department of Orthopaedic Surgery, School of Medicine, Kyung Hee University, Seoul 02447, Republic of Korea; 3 Department of Orthopaedic Surgery, Hallym Sacred Heart University Hospital, Hallym University, Anyang-si 13496, Republic of Korea

**Keywords:** high tibial osteotomy, popliteal cyst, Baker’s cyst, osteoarthritis, partial meniscectomy

## Abstract

**Introduction:** Popliteal cysts (PCs) are occasionally accompanied by knee osteoarthritis (OA) and varus malalignment. However, whether concomitant arthroscopic excision of PCs with medial open-wedge high tibial osteotomy (MOWHTO) improves the osteoarthritic environment remains unclear. Therefore, this study assessed serial changes in C-size, medial meniscus extrusion (MME), and cartilage status for up to 2 years following an MOWHTO. **Methods:** This study retrospectively used serial magnetic resonance imaging (MRI) evaluations to assess 26 consecutive patients who underwent MOWHTO. Of the 26 patients, six with preoperative PCs were included. Based on the arthroscopic findings at the time of the MOWHTO, concomitant meniscal and chondral lesions, and whether or not partial meniscectomy was performed, were evaluated. All patients underwent second-look arthroscopy with plate removal 2 years postoperatively. The PC size, MME, and cartilage sub-scores in the medial compartment of the whole-organ MRI score (WORMS) were assessed by serial MRI preoperatively and at 3, 6, 18, and 24 months postoperatively. The recurrence of PCs and clinical outcomes, including the Rauschning–Lindgren grade, were also evaluated when serial MRI was performed. Moreover, changes in cartilage status were assessed using two-stage arthroscopy. **Results:** All patients underwent concomitant partial meniscectomy for medial meniscal tears in the posterior horn. A significant decrease in the mean size of preoperative PCs (27.4 ± 22.3 mm) was noted from 3 months postoperatively (8.7 ± 7.6 mm, *p* = 0.018), and thereafter. The mean size of PCs further decreased with time until 2 years (1.5 ± 4.0 mm, *p* = 0.018) following an MOWHTO with partial meniscectomy. Moreover, significant improvements in the MME and WORMS values were noted from 3 to 24 months postoperatively. Meanwhile, no PC recurrence occurred during the follow-up period and the preoperative Rauschning–Lindgren grade improved significantly with time after MOWHTO (*p* = 0.026). Furthermore, the two-stage arthroscopic assessments showed significant improvements in ICRS grade in the medial femoral condyle (*p* = 0.038). **Conclusions:** After an MOWHTO with partial meniscectomy, PCs decreased with time up to 2 years postoperatively; no recurrence occurred during the follow-up period, although cyst excision was not concomitantly performed. Furthermore, the reduction in PCs corresponded with improvements in MME and chondral lesions in the knee joint following the MOWHTO.

## 1. Introduction

Popliteal cysts (PCs) are occasionally encountered during physical examinations or advanced imaging evaluations in patients with knee osteoarthritis (OA), with reported incidence rates ranging from 19% to 41% [1,2]. Previous studies have elucidated that PCs resulting from an osteoarthritic environment, including chondral lesions and torn menisci, develop joint effusion and increased intra-articular pressure, causing synovial fluid leakage into the popliteal area via a deficiency of the posterior capsule [1,2,3].

Although most patients with PCs are asymptomatic, some present symptoms requiring treatment such as a palpable mass, pain, and limited range of motion (ROM) [2]. Recently, the arthroscopic excision of PCs, which has a low recurrence rate [4,5], has been the treatment of choice for symptomatic patients who are refractory to conservative treatment. Patients undergoing medial open-wedge high tibial osteotomy (MOWHTO) have symptomatic knee osteoarthritis with varus malalignment; thus, these patients are theoretically thought to have a higher incidence of PCs, considering the osteoarthritic environment of the knee joint and excessive intra-articular pressure in the medial compartment [5,6]. However, it is unclear as to whether the concomitant arthroscopic excision of PCs is necessary for patients with PCs undergoing an MOWHTO.

As concomitantly performing arthroscopic excision of PCs with MOWHTO requires a longer operation time, an additional posterior approach, and carries a risk of complications, patients scheduled for MOWHTOs with preoperative, symptomatic PCs are of great concern to surgeons [4,5]. Meanwhile, the MOWHTO improves the osteoarthritic environment by regenerating chondral lesions [7,8], decreasing the extrusion of torn medial menisci [9,10,11], and reducing intra-articular pressure in the medial compartment [12]; thus, this procedure may itself decrease PCs without cyst excision. Moreover, as tears of the medial meniscal posterior horn are frequently observed in patients undergoing MOWHTOs [11,13], concomitant partial meniscectomy at the medial meniscal posterior horn also decreases PCs, because it leads to the opening of the valvular mechanism of PCs [3,14]. However, no existing studies have investigated the natural progression of PCs after an MOWHTO without cyst excision.

Thus, the current study performed serial magnetic resonance imaging (MRI) evaluations to assess the serial changes, for up to 2 years, in PCs following an MOWHTO without cyst excision. We hypothesized that PCs would decrease following an MOWHTO for up to 2 years, in accordance with concurrent improvements in chondral lesions and torn menisci.

## 2. Methods

### 2.1. Patients

Following local institutional review board approval, we retrospectively reviewed prospectively collected data of 26 consecutive patients between December 2016 and March 2018, which have not been previously reported on, to evaluate the serial changes in PCs after an MOWHTO without cyst excision. This study was a case series into which patients were prospectively enrolled and included patients who underwent MOWHTOs based on the following criteria: (1) symptomatic medial compartment OA with varus malalignment of >5°; (2) age < 70 years; (3) OA of Kellgren–Lawrence (K–L) grade 1–3; (4) a minimum of 2 years of follow-up; and (5) the presence of a PC. Patients were excluded if they had prior meniscal surgery, such as meniscus repair or meniscectomy, PC excision, prior ligament surgery, or a history of ligament injuries. PCs were defined as extra-articular cysts originating from the joint and categorized as Baker’s cysts (BCs) or popliteal ganglion cysts, depending on their location and pathology [1,15]. BCs and popliteal ganglion cysts result from distention of the gastrocnemio-semimembranosus bursa and degeneration of the posterior joint capsule or tendons of the medial and lateral heads of the gastrocnemius, respectively [1,2,15]. The presence and type of PCs were determined by two musculoskeletal radiologists. This study was approved by Kyung-Hee University Hospital at Gandong (KHNMC 2022-01-023). The study was conducted in accordance with the Helsinki declaration. Local institutional review board (Kyung-Hee University Hospital at Gandong) approval was obtained before the study. The study was granted an exemption from requiring informed consent by an ethics committee (Kyung-Hee University Hospital at Gandong, (KHNMC 2022-01-023)).

### 2.2. Surgical Technique and Rehabilitation Protocol

A single senior surgeon (KIK) performed the MOWHTO in all cases. All patients underwent arthroscopic evaluation at the time of the MOWHTO and no cartilage restorations, meniscal repairs, or cyst excisions were concomitantly performed. Partial meniscectomy was performed only when degenerative flap tears or root tears of the medial meniscal posterior horn were observed. The detailed surgical technique and MOWHTO procedure have been previously described [8,16,17]. Following the arthroscopic procedure, a biplanar osteotomy was performed. The correction angle was determined based on preoperative planning using the Miniaci method [18] and was adjusted by targeting the mechanical axis close to 62% of the tibial plateau from the medial edge, depending on the arthroscopic cartilage status [8]. Fixation of the osteotomy site was performed using a medial locked-plate system (Tomofix; Depuy Synthes, Solothurn, Switzerland). The opening gap was filled when the gap width exceeded 12 mm. On postoperative day 1, range-of-motion exercise was initiated and partial weight-bearing with a crutch was allowed for 6 weeks. Full weight-bearing was allowed 6 weeks after surgery. Compressive or aggressive physical therapy was contraindicated in all patients. Furthermore, it cannot be concluded that the postoperative physical therapy or rehabilitation protocols did not influence the reduction of popliteal cysts following the MOWHTO and partial meniscectomy. Plate removal and second-look arthroscopic evaluations were performed in all patients, 2 years after the MOWHTO.

### 2.3. Serial MRI Assessments

Changes in PCs were evaluated based on serial MRI evaluations performed at 3, 6, 18, and 24 months from the preoperative MRI evaluation. All patients underwent the same 3.0-T MRI protocol (Philips, Amsterdam, The Netherlands) at all time periods. The PC sizes were measured at the longest dimension in the superior–inferior length of the sagittal plane [15,19]. During the serial MRI evaluations, postoperative decrease, disappearance, or recurrence of the PCs were evaluated.

For the serial evaluation of the medial meniscus following the MOWHTO, medial meniscus extrusion (MME) was measured consecutively before the MOWHTO, and at 3, 6, 18, and 24 months postoperatively, by MRI [15,20]. The MME values were measured from the medial margin of the tibial plateau except for the osteophyte to the external border of the medial meniscus on the T1 coronal image showing maximal extrusion [15]. For serial evaluation of the cartilage status in the medial compartment, the whole-organ MRI score (WORMS) was also evaluated [21]. Within the WORMS, the sub-score of the cartilage in the medial compartment was evaluated [21]. The scores ranged from 0 to 30, with higher scores indicating a worse cartilage status [21].

### 2.4. Evaluations

The radiological evaluation included assessments of the K–L grade [22], hip–knee–ankle angle (HKAA) [23], medial proximal tibial angle (MPTA) [24], posterior tibial slope angle (PTSA) [23], and correction angle [25]. Clinical evaluations included the range of motion (ROM), total Western Ontario and McMaster Universities Osteoarthritis Index (WOMAC) score, Knee Injury and Osteoarthritis Outcome Score (KOOS), and Rauschning–Lindgren grade [26]. Postoperative complications, including infection, deep vein thrombosis, or fixation failure and failure, defined as conversion to arthroplasty or revisional MOWHTO, were evaluated. For the arthroscopic assessment, articular cartilage was evaluated at the time of MOWHTO and second-look arthroscopy according to the International Cartilage Repair Society (ICRS) grade [27] was conducted. Moreover, the grade of cartilage regeneration of the medial femoral condyle (MFC) and medial tibial plateau (MTP) following the MOWHTO was classified based on the macroscopic staging system by Koshino et al. [28]. Two musculoskeletal radiologists blindly evaluated radiologic variables, and the discrepancy was resolved by discussion.

### 2.5. Statistical Analyses

IBM SPSS Statistics for Windows, version 23.0 (IBM Corp., New York, NY, USA) was used for the statistical analysis, with *p* < 0.05 considered statistically significant. Continuous data are presented as means, standard deviations, and ranges. Categorical data are presented as frequencies and percentages. Descriptive statistics were used to assess patient demographics. Wilcoxon rank-sum tests were used to compare preoperative and postoperative outcomes. The two-way false discovery rate was determined for multiple comparisons; thus, the adjusted *p*-value was calculated accordingly [29]. The reliability of all measurements was analyzed using intra-class correlation coefficients. The reliabilities of the radiologic values were assessed with inter- and intra-class correlation coefficients, which ranged from 0.85 to 0.96 and from 0.82 to 0.93, respectively, indicating high agreement between observers [30,31].

## 3. Results

Of the 26 consecutive patients, this study enrolled six (23.1%) who had PCs before an MOWHTO. Of these six PCs, five were BCs, while one was a popliteal ganglion cyst. All the patients completed serial MRI evaluations over 2 years. The mean HKAA and MPTA were significantly corrected after the MOWHTOs (*p* = 0.018). The mean total WOMAC score and KOOS, except for the sports subscale, were also significantly improved at the 2-year follow-up, compared to the preoperative scores (Table 1). No complications or failures were observed.

**Figure 1:**
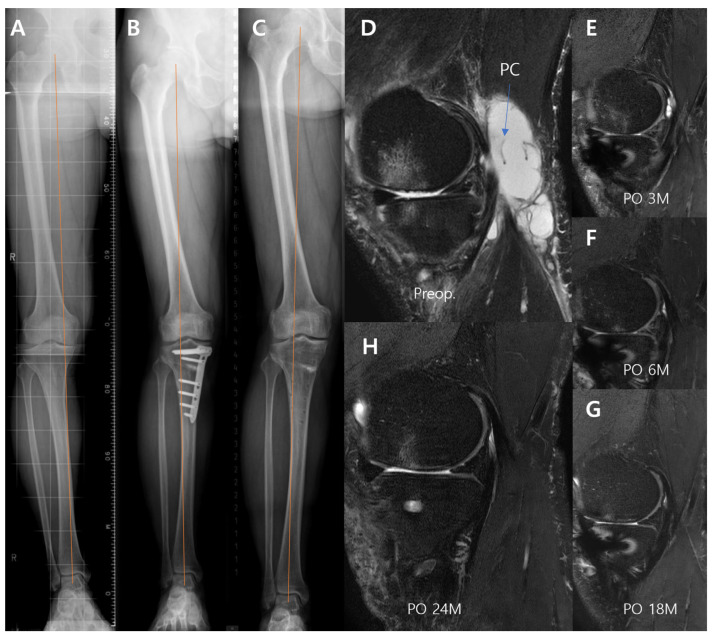
Changes in the simple radiographs and popliteal cyst in MRI after medial open-wedge high tibial osteotomy (MOWHTO): preoperative medial compartment osteoarthritis (OA) with varus alignment (**A**); right knee of 62-year-old male patient is corrected to valgus alignment via MOWHTO (**B**); maintained well at postoperative 2 years after plate removal (**C**); preoperative popliteal cyst (**D**); cyst decreased with time after MOWHTO, observed through serial MRI follow-up to postoperative 24 months (**E**–**H**). PC, popliteal cyst; preop., preoperative; PO, postoperative.

A postoperative decrease in PCs was noted in all patients at 3 months after the MOWHTOs. The mean size of the preoperative PCs (27.4 ± 22.3 mm) was significantly decreased after 3 months, postoperatively (8.7 ± 7.6 mm, *p* = 0.018); thereafter, the mean PC size further decreased with time until 2 years (1.5 ± 4.0 mm, *p* = 0.018) following the MOWHTO (Figure 1). Furthermore, the preoperative Rauschning–Lindgren grade significantly improved with time after the MOWHTO (*p* = 0.026) (Table 2). However, there were no recurrences of PCs.

Regarding the serial changes in the medial meniscus and cartilage status in the medial compartment on MRI evaluation, significant improvements in MME and WORMS values were noted from 3 months, postoperatively. The mean MME and WORMS values further improved with time, following the MOWHTO (Table 3).

Based on the arthroscopic findings at the time of the MOWHTO, all patients had medial meniscal tears, including medial meniscal posterior horn root tear (50% of cases) and degenerative flap tears at the medial meniscal posterior horn (50% of cases); thus, all patients underwent a concomitant partial meniscectomy. Moreover, all the patients had chondral defects in the medial compartment. Significant improvements in ICRS grades were observed in the MFC based on the two-stage arthroscopic assessments (*p* = 0.038). Total regeneration of the cartilage in the MFC and MTP groups was observed in three (50%) and one (16.7%) cases, respectively (Table 4).

## 4. Discussion

The results of the current study demonstrated decreased PCs following an MOWHTO with partial meniscectomy but without cyst excision. This reduction was observed without recurrence from 3 months to 2 years, postoperatively, based on serial MRI evaluations. Moreover, significant improvements in the meniscus and cartilage were concurrently observed in terms of MME and WORMS from 3 months following the MOWHTO, which corresponded to the decreased PCs.

Arthroscopic correction of intra-articular pathology without cyst excision was previously attempted to abolish PCs [3,5,32]. Rupp et al. reported on the successful treatment of PCs with low-grade chondral lesions or meniscal tears via the arthroscopic correction of intra-articular pathology [32]. Nevertheless, PCs with high-grade chondral lesions were not eliminated after the microfracture technique because synovial effusion remained in the joint despite the procedure [32]. As PCs frequently accompany knee OA with high-grade chondral lesions, subsequent studies have been performed to directly access and excise PCs to decrease the recurrence rates, owing to advances in arthroscopic surgical techniques [4,15,33]. However, there remains a paucity of research on the effect of MOWHTOs on PCs, as varus malalignment is a crucial factor for intra-articular pathology in knee OA [12]. Therefore, we performed serial MRI evaluations to assess the fate of PCs after MOWHTOs in consecutive patients with knee OA, as correction of the varus malalignment seemed effective for the shrinkage of PCs. The results showed that the PCs decreased in size over 2 years following the MOWHTO, without recurrence. Moreover, three of the six PCs were finally eliminated.

Considering the effectiveness of MOWHTO, the results of the current study are not unexpected, as the MOWHTO shifts the weight-bearing load to the unaffected compartment to decrease intra-articular pressure in the medial compartment [12], which might lead to PC shrinkage. Moreover, owing to the biomechanical correction, the MOWHTO ameliorates intra-articular pathology in the medial compartment, including torn menisci and chondral lesions [7,8,9,10]. Recent studies demonstrated that the MOWHTO improved preoperative MME, which indicates degeneration of the medial meniscus and is a risk factor for the aggravation of knee OA [9,10]. The results of the current study were consistent with those of recent studies reporting improved MME 3 months following an MOWHTO compared to preoperative values; moreover, the improvement was maintained for >24 months, postoperatively [9,10]. Additionally, multiple studies have reported on the regeneration of degenerated articular cartilage in the medial compartment after an MOWHTO alone [7,8,34,35]. In the current study, cartilage evaluation by WORMS through serial MRI and two-stage arthroscopic findings showed significant improvements in the chondral lesions in the medial compartment after an MOWHTO, consistent with previous reports [7,8,34,35]. Taken together, these data indicate that MOWHTOs without cyst removal can decrease PCs owing to the improvements in MME and chondral lesions as well as to the decompression of the medial compartment. Furthermore, biomechanical correction of the osteoarthritic environment with improvement of the intra-articular disorder can minimize the risk of recurrent effusions, which is crucial for PCs.

Previous studies reported that the presence of the valvular mechanism of the posteromedial capsule and effusion create a unidirectional flow of synovial fluid from the joint cavity to PCs, which is a fundamental factor for the persistence of PCs [5,14,36]. Recent studies have emphasized the direct accession and excision of the posteromedial synovial fold, which functions as a valvular mechanism, to avoid PC recurrence [4,15,36,37,38,39]. However, the results of the present study demonstrated decreased PC size and no recurrence after only an MOWHTO without excision of the valvular mechanism. Notably, all patients in the current study underwent an MOWHTO with partial meniscectomy for degenerative root or flap tears of the medial meniscal posterior horn. The performance of a partial meniscectomy to tears in the medial meniscal posterior horn would have resulted in the correction of the valvular mechanism between the joint cavity and PCs, as previous studies reported a thinner and more fragile septum between the two structures just behind the posterior horn [3,14]. Thus, the effects of a partial meniscectomy on medial meniscal posterior horn tears in addition to MOWHTO potentially led to a reduction in PCs without recurrences. The results of the current study provide valuable information to surgeons for patients scheduled for MOWHTO with preoperative, symptomatic PCs to avoid additional operation time and complications due to PC excision via the arthroscopic posterior approach [4,37]. Particularly, the risk of complications, including hematoma, pain, swelling, portal-site infection, and neurovascular damage, is increased when wall resection of PCs is performed instead of wall preservation [4,37,40]. Based on these results, patients with symptomatic knee OA, varus malalignment, and PCs can be treated with an MOWHTO without PC excision in addition to concomitant partial meniscectomy in cases with concurrent medial meniscal posterior horn tears.

Despite the informative results of the current study, there are several limitations. First, the study had a retrospective design and small sample size. As the current study was a retrospective analysis of prospectively collected data, only six patients (23.1%) with PCs were available for analysis among the 26 patients in the entire cohort. Second, not all patients with PCs in the current study had symptoms of PCs because the original study was not intended to evaluate serial changes in PCs following an MOWHTO. Moreover, the mean size of the PCs was relatively smaller than those of other studies evaluating symptomatic PCs [4,15,40]. However, the current study was meaningful in that it performed consecutive MRI evaluations of PCs following MOWHTOs up to 2 years, postoperatively, as no other studies have applied serial MRI evaluations to report on the natural progression of PCs after an MOWHTO. Third, the current study is still limited with respect to confirming the results and needs more robust evidence to prove the results because this topic has not been covered previously. Finally, because we evaluated East Asian patients, the demographic characteristics of the current study should be noted before extrapolating our findings to other populations, as a more frequent varus malalignment and a marked female predominance in the knee OA population might be differences that require consideration [41]. Meanwhile, we initially intended to conduct a subsequent study comparing the efficacy of an MOWHTO alone versus an MOWHTO combined with PC excision. However, our findings indicate that MOWHTOs with a partial meniscectomy can reduce the size of PCs without direct excision. Consequently, we plan a follow-up study with a larger sample size and a more robust design to validate these results.

## 5. Conclusions

After the MOWHTO with partial meniscectomy, PCs decreased over time for up to 2 years, postoperatively. Moreover, no recurrence occurred during the follow-up period, although cyst excision was not concomitantly performed. Furthermore, the reduction in PCs corresponded with improvements in MME and chondral lesions in the knee joint following the MOWHTO.

## Figures and Tables

**Table 1:** Patient characteristics and comparisons of radiologic and clinical outcomes, preoperatively and postoperatively ^α^.

	Preoperative	Postoperative	*p* Value ^‡^
Demographic data			
Age, years	61.7 ± 4.5 (54–66)	-	-
Sex, male/female	2/4	-	-
BMI, kg/m^2^	24.5 ± 2.7 (21.2–29.4)	-	-
Side, right/left	4/2	-	-
Follow-up, months		42.3 ± 9.3 (30–49)	
Radiologic data ^‡^			
K–L grade, (2/3/4)	2/4/0	1/5/0	0.564
HKAA ^†^, °	−7.0 ± 1.5 (−9.3–−5.8)	2.2 ± 1.4 (0–4.0)	0.018 *
MPTA, °	83.8 ± 1.4 (81.2–85.6)	93.6 ± 1.7 (91.0–95.8)	0.018 *
PTSA, °	8.7 ± 2.3 (6.1–11.3)	8.7 ± 2.6 (4.7–13.1)	0.866
Correction angle, °	-	10.2 ± 1.5 (8.5–13.0)	-
Clinical outcomes ^‡^			
ROM, °	135.7 ± 8.4 (120–145)	141.4 ± 2.4 (140–145)	0.109
WOMAC total score	46.3 ± 19.9 (18.4–71.7)	18.9 ± 13.1 (4.8–40.2)	0.043 *
KOOS symptom	60.2 ± 22.3 (17.9–82.1)	82.1 ± 9.9 (71.4–96.4)	0.042 *
KOOS pain	58.3 ± 25.7 (22.2–86.1)	77.4 ± 10.7 (61.1–86.1)	0.028 *
KOOS ADL	63.7 ± 19.7 (33.8–91.2)	81.5 ± 8.9 (64.7–92.7)	0.045 *
KOOS sports	27.9 ± 19.5 (5–65.0)	32.9 ± 10.4 (20.0–50.0)	0.344
KOOS QoL	31.3 ± 14.0 (18.8–50.0)	50.9 ± 6.7 (43.8–62.5)	0.034 *

^α^ Values are presented as numbers or means ± standard deviation (range). BMI, body mass index; K–L, Kellgren–Lawrence; HKAA, hip–knee–ankle angle; MPTA, medial proximal tibial angle; PTSA, posterior tibial slope angle; ROM, range of motion; WOMAC, Western Ontario and McMaster Universities Osteoarthritis score; KOOS, Knee Injury and Osteoarthritis Outcome Score; ADL, activities of daily living; QoL, quality of life. ^†^ A negative value indicates varus alignment. ^‡^ Wilcoxon signed-rank test was performed to compare the preoperative and postoperative values. * Significance was set at *p* < 0.05. °, degree.

**Table 2:** Serial evaluations of popliteal cysts following MOWTHO during a 2-year follow-up period ^α^.

	Preoperative	Postoperative			
		3 months	6 months	18 months	24 months
Patients with decreased PC (%)		6 (100)	6 (100)	6 (100)	6 (100)
Patients with disappeared PC (%)		2 (33.3)	3 (50.0)	3 (50.0)	3 (50.0)
Cyst size, mm	27.4 ± 22.3 (10.4–75.2)	8.7 ± 7.6 (0–19.3)	6.7 ± 7.5 (0–17.7)	3.8 ± 6.6 (0–15.7)	1.5 ± 4.0 (0–10.6)
*p* value ^†^		0.018 *	0.018 *	0.018 *	0.018 *
Rauschning and Lindgren grade, 0/1/2/3	0/1/4/1	2/3/1/0	3/3/0/0	5/1/0/0	5/1/0/0
*p* value ^†^		0.026 *	0.026 *	0.026 *	0.026 *
Recurrence		0	0	0	0

^α^ Values are presented as mean ± standard deviation (range) and number (%). MOWHTO, medial open-wedge high tibial osteotomy; PC, popliteal cyst. ^†^ *p*-value adjusted for multiple comparisons using the false discovery rate (FDR). * Statistical significance was set at *p* < 0.05.

**Table 3:** Serial MRI changes in associated meniscus and cartilage pathology following MOWHTO during a 2-year follow-up period ^α^.

	Preoperative	Postoperative			
		3 months	6 months	18 months	24 months
Medial meniscus tear					
MME, mm	7.2 ± 0.4 (6.7–8.0)	6.5 ± 0.6 (5.6–7.6)	5.9 ± 0.7 (5.0–6.9)	6.0 ± 1.2 (4.5–7.6)	5.8 ± 0.9 (5.0–6.9)
*p* value ^†^		0.046 *	0.036 *	0.046 *	0.036 *
Cartilage					
WORMS score ^‡^	14.2 ± 3.3 (11–20.5)	13.0 ± 3.7 (9–20.5)	11.6 ± 2.3 (9–15)	8.5 ± 2.8 (5–12)	7.5 ± 2.4 (4–11)
*p* value ^†^		0.041 *	0.024 *	0.024 *	0.024 *

^α^ Values are presented as mean ± standard deviation (range). MME, medial meniscus extrusion; WORMS, whole-organ magnetic resonance imaging. ^†^ *p*-value adjusted for multiple comparisons using the false discovery rate (FDR). ^‡^ The WORMS sub-score of cartilage in the medial compartment was evaluated and ranged from 0 to 30. * Statistical significance was set at *p* < 0.05.

**Table 4:** Arthroscopic findings of the associated meniscus and cartilage pathology, preoperatively and postoperatively ^α^.

	Preoperative	Postoperative	*p* Value ^‡^
Medial meniscus tear	6 (100)	0	-
MMRT	3 (50.0)	-	-
Degenerative flap tear	3 (50.0)	0	-
Partial meniscectomy	6 (100)	0	
Lateral meniscus tear	0	0	-
Cartilage	6 (100)		
ICRS grade			
MFC, grade 1/2/3/4	0/0/2/4	2/2/1/1	0.038 *
MTP, grade 1/2/3/4	0/2/1/3	1/2/2/1	0.102
Koshino’s macroscopic grade for cartilage regeneration ^†^			
MFC, grade A/B/C	-	2/1/3	-
MTP, grade A/B/C	-	1/4/1	-

^α^ Values are presented as numbers (%). MMRT, medial meniscus root tear; ICRS, International Cartilage Repair Society; MFC, medial femoral condyle; MTP, medial tibial plateau. ^†^ Grades A, B, and C indicate no regeneration, partial regeneration, and total regeneration, respectively. ^‡^ Wilcoxon signed-rank test was performed to compare the preoperative and postoperative values. * Significance was set at *p* < 0.05.

## Data Availability

Data and materials are available on request to the corresponding author.

## List of Abbreviations

BC	Baker’s cyst
HKAA	hip-knee-ankle angle
ICRS	International Cartilage Repair Society
KOOS	Knee Injury and Osteoarthritis Outcome Score
MFC	medial femoral condyle
MME	medial meniscus extrusion
MPTA	medial proximal tibial angle
MOWHTO	medial open-wedge high tibial osteotomy
MRI	magnetic resonance imaging
MTP	medial tibial plateau
OA	osteoarthritis
PC	popliteal cyst
PTSA	posterior tibial slope angleROM: range of motion
WOMAC	Western Ontario and McMaster Universities Osteoarthritis Index
WORMS	whole-organ MRI score
